# Dietary Intake Is Related to Multifactor Cardiovascular Risk Score in Obese Boys

**DOI:** 10.3390/healthcare2030282

**Published:** 2014-07-23

**Authors:** Tracy L. Schumacher, Tracy L. Burrows, Dylan P. Cliff, Rachel A. Jones, Anthony D. Okely, Louise A. Baur, Philip J. Morgan, Robin Callister, May M. Boggess, Clare E. Collins

**Affiliations:** 1 School of Health Sciences, Faculty of Health and Medicine, University of Newcastle, Newcastle, NSW 2308, Australia; E-Mails: Tracy.Schumacher@uon.edu.au (T.L.S.); Tracy.Burrows@newcastle.edu.au (T.L.B.); May.Boggess@gmail.com (M.M.B.); 2 Priority Research Center in Physical Activity and Nutrition, University of Newcastle, Newcastle, NSW 2308, Australia; E-Mails: Philip.Morgan@newcastle.edu.au (P.J.M.); Robin.Callister@newcastle.edu.au (R.C.); 3 Early Start Research Institute, School of Education, Faculty of Social Science, University of Wollongong, Wollongong, NSW 2522, Australia; E-Mails: Dylanc@uow.edu.au (D.P.C.); Rachelj@uow.edu.au (R.A.J.); Tokley@uow.edu.au (A.D.O.); 4 The Children’s Hospital at Westmead Clinical School, Sydney Medical School, University of Sydney, Sydney, NSW 2145, Australia; E-Mail: Louise.Baur@health.nsw.gov.au; 5 School of Education, Faculty of Education and Arts, University of Newcastle, Newcastle, NSW 2308, Australia; 6 School of Biomedical Science and Pharmacy, Faculty of Health and Medicine, University of Newcastle, Newcastle, NSW 2308, Australia; 7 School of Mathematical and Statistical Sciences, Arizona State University, Tempe, AZ 85287, USA

**Keywords:** cardiovascular risk, childhood, obesity, diet, nutrition

## Abstract

Cardiovascular disease (CVD) originates in childhood and early identification of risk factors provides an early intervention opportunity. The aim was to identify children at higher risk using a CVD risk score, developed from factors known to cluster in childhood. Risk was scored as very high (≥97.5th centile), high (≥95th), moderate (≥90th) or threshold (<90th) using normal pediatric reference ranges for 10 common biomedical risk factors. These were summed in a multifactor CVD risk score and applied to a sample of 285 observations from 136 overweight Australian children (41% male, aged 7–12 years). Strength of associations between CVD risk score and individual biomedical and dietary variables were assessed using univariate logistic regression. High waist circumference (Odds Ratio: 5.48 [95% CI: 2.60–11.55]), body mass index (OR: 3.22 [1.98–5.26]), serum insulin (OR: 3.37 [2.56–4.42]) and triglycerides (OR: 3.02 [2.22–4.12]) were all significantly related to CVD risk score. High intakes of total fat (OR: 4.44 [1.19–16.60]), sugar (OR: 2.82 [1.54–5.15]) and carbohydrate (OR 1.75 [1.11–2.77]) were significantly related to CVD risk score in boys only. This multifactor CVD risk score could be a useful tool for researchers to identify elevated risk in children. Further research is warranted to examine sex-specific dietary factors related to CVD risk in children.

## 1. Introduction

The World Health Organization (WHO) reported in 2008 that 17.3 million deaths worldwide were due to cardiovascular disease (CVD) [1]. A major contributor to CVD is atherosclerosis which is a dynamic process that can begin in childhood and develop or regress, depending on the presence or absence of a range of risk factors, including obesity, inflammation, hyperglycemia, hypertension and hyperlipidemia [2].

The International Obesity Taskforce estimates that approximately 40–50 million school aged children are obese [3]. Obese children are at high risk of becoming obese adults, especially if their parents are also obese [4]. Abdominal obesity in children is associated with low grade inflammation [5], a significant contributor to the development of atherosclerosis [2]. Both body mass index (BMI) and waist circumference (WC) correlate with intra-abdominal fat in primary school aged children [6] and are used as clinical measures to identify CVD risk [7]. Obese children are also at increased risk of hypertension and dyslipidemia as they age [8].

A number of risk factors cluster within both adults and children [9,10]. Those most commonly identified in overweight and obese children are elevated fasting serum insulin and glucose [5,11,12], high blood pressure [5,11,13,14], raised triglycerides [5,11,12,14], total [5,14] and LDL-cholesterol [11,12,14]; and low HDL-cholesterol [11,12,13,14]. There are a number of accepted standard reference ranges used to quantify these risk factors in adults [15,16], with age- and sex-specific reference values recently developed for children [17,18] using population data from France [18]. The Framingham method for assessing 30 year risk in adults includes age, sex, systolic blood pressure (SBP), anti-hypertensive medications, smoking, presence of diabetes, total and HDL cholesterol [19]. Although scoring methods for determining CVD risk in children exist [20], a straightforward method of assessing and describing a gradation of risk in overweight children by comparing individual clinical data to population-derived percentile bands could assist in estimating future risk of CVD. Therefore the aims of this study were to; (1) develop a multifactor CVD risk score using these reference ranges for pediatric researchers; (2) assess its application in overweight and obese pre-pubertal children; (3) examine the difference in dietary intake between high *versus* low category CVD risk scores; and (4) to examine the strength of associations between individual anthropometric and biomedical risk factors in both boys and girls.

## 2. Experimental Section

Data for the current study were obtained for secondary analysis from a primary study: the Hunter Illawarra Kids Challenge Using Parent Support (HIKCUPS) randomized controlled trial. Overweight and obese children aged 5 to10 years were recruited between 2005–2006 and followed up for a period of 24 months from baseline. The children were from predominantly middle-class families with English-speaking backgrounds. The original purpose of this study was to determine the efficacy of three different weight loss interventions in overweight children. Detailed methods for the study and outcomes are described elsewhere [21,22,23,24,25]. Data were collected at up to four time points (baseline, 6-, 12-, and 24-month follow-up). The current analyses included those children with complete blood, dietary and anthropometric data sets at individual time points for children aged seven years or older at the time of the first measurement, reflecting the minimum age of the reference values used [18]. This included 285 observations on 136 individuals, comprised of 112 baselines measures with 79, 57 and 37 measures respectively from the remaining time points. This provides data from children at a wider range of ages and with changing weight status. Written informed consent was obtained from the parents, the trial was registered with the U.S. National Center for Clinical Trials (00107692) and approval was obtained from the human research ethics committees of the University of Newcastle and University of Wollongong.

### 2.1. Anthropometry and Serum Sampling

Participants were weighed in light clothing to the nearest 0.1 kg using Tanita HD646 scales (Tanita Corporation, Chicago, IL, USA). Height was measured to the nearest 0.1 cm using PE87 portable stadiometers (Mentone Educational Center, Victoria, Australia). Waist circumference (WC) was measured at the midpoint between the lower costal border and the iliac crest [26]. Blood pressure was measured using an automated monitor (Critikon, Tampa, FL, USA) under standardized conditions. Blood samples were collected for glucose, insulin and blood lipid concentrations from the children after an overnight fast by trained phlebotomists and analyzed at a single accredited pathology service (National Association of Testing Authorities, Newcastle, Australia).

### 2.2. HIKCUPS CVD Risk Score

BMI, WC, systolic blood pressure (SBP), diastolic blood pressure (DBP), serum HDL, LDL and total cholesterol, serum triglycerides, serum fasting glucose and serum fasting insulin were used to develop a multifactor CVD risk score based on the premise that elevated childhood values for these individual measures have been shown to predict later CVD in adulthood [27,28,29]. Values greater than or equal to the top tenth percentile of normal reference ranges were used to identify those at the greatest risk and to identify risk score cut-points. This is in a manner comparable to those used in the International Diabetes Federation (IDF) definition of pediatric metabolic syndrome [30] and the National Heart, Lung and Blood Institute [31] for identification of elevated plasma lipids, with a scoring similar to that used by Bergmann *et al.* [32] to identify individuals at higher odds of CVD risk factors. BMI reference values were from the US Centers for Disease Control and Prevention age and sex specific growth charts [33] and those for all other measures were developed from a healthy population by Mellerio *et al*. [18], although in that study WC was measured at the umbilicus. The 90th, 95th and 97.5th percentile values for each measure for children aged 7–11 years are provided in Table 1.

**Table 1:** Percentiles of clinical and biochemical cardiovascular disease (CVD) risk score factors by age and sex.

Age	7 Years			8 Years			9 Years			10 Years			11 Years		
Percentile	**90**	**95**	**97.5**	**90**	**95**	**97.5**	**90**	**95**	**97.5**	**90**	**95**	**97.5**	**90**	**95**	**97.5**
**Boys**															
BMI *	17.7	18.8	19.5	18.3	19.7	20.9	20.4	22.3	23.1	20.1	22	23.1	21.7	23	25
WC	61.4	63.6	65.5	63.7	66	68.1	66	68.5	70.8	68.4	71.1	73.6	70.9	73.7	76.4
Systolic BP	114	118	122	116	120	124	118	123	127	121	125	129	124	128	132
Diastolic BP	70	73	75	71	74	76	71	74	77	72	75	78	73	76	79
F. glucose	4.87	5	5.11	4.96	5.09	5.21	5.04	5.17	5.3	5.11	5.24	5.37	5.16	5.3	5.42
Insulin	5.99	7.05	8.09	7.16	8.45	9.73	8.47	10	11.6	9.88	11.8	13.6	11.3	13.5	15.7
Total chol	5.5	5.84	6.15	5.52	5.85	6.15	5.53	5.85	6.14	5.51	5.83	6.11	5.47	5.78	6.06
LDL	3.79	4.07	4.33	3.75	4.03	4.29	3.7	3.99	4.24	3.66	3.94	4.2	3.62	3.9	4.15
HDL	1.94	2.07	2.19	1.95	2.08	2.2	1.95	2.08	2.2	1.94	2.07	2.18	1.91	2.03	2.15
Triglycerides	0.72	0.81	0.91	0.8	0.91	1.03	0.87	1.01	1.15	0.94	1.1	1.27	1.01	1.19	1.38
**Girls**															
BMI	17.8	19.2	20	18.9	19.9	21.6	20.1	22.9	24.3	21	23.2	23.8	22.4	24.7	27
WC	61.2	63.6	66	63.9	66.6	69.2	66.5	69.4	72.2	68.9	72	75	71.1	74.4	77.6
Systolic BP	112	116	120	115	118	122	117	121	124	119	123	127	121	125	129
Diastolic BP	70	73	75	71	74	76	71	74	77	72	75	78	73	76	79
F. glucose	4.81	4.97	5.11	4.88	5.04	5.18	4.96	5.11	5.24	5.03	5.17	5.3	5.08	5.22	5.34
Insulin	7.59	9.18	10.8	8.55	10.2	12	9.67	11.5	13.4	11.1	13.1	15.2	13	15.3	17.7
Total chol.	5.77	6.15	6.5	5.67	6.05	6.39	5.58	5.95	6.29	5.51	5.87	6.2	5.45	5.8	6.13
LDL	4.1	4.47	4.82	3.98	4.34	4.69	3.88	4.23	4.56	3.78	4.13	4.46	3.71	4.05	4.37
HDL	1.78	1.9	2.02	1.82	1.94	2.06	1.82	1.95	2.07	1.82	1.94	2.06	1.8	1.93	2.04
Triglycerides	0.84	0.95	1.07	0.92	1.06	1.19	1.00	1.15	1.29	1.07	1.23	1.38	1.13	1.3	1.47
Percentile	**10**	**5**	**2.5**	**10**	**5**	**2.5**	**10**	**5**	**2.5**	**10**	**5**	**2.5**	**10**	**5**	**2.5**
**Boys**															
HDL	1.23	1.15	1.09	1.23	1.16	1.09	1.23	1.16	1.09	1.23	1.15	1.09	1.21	1.13	1.07
**Girls**															
HDL	1.11	1.03	0.98	1.13	1.05	1	1.13	1.06	1	1.13	1.06	1	1.12	1.05	0.99

* BMI is 97th percentile not 97.5th percentile. BMI values are taken from CDC reference ranges [33] and waist circumference, blood pressure, fasting glucose, insulin, triglycerides, LDL, HDL and total cholesterol are those developed by Mellerio [18]. BMI—Body Mass Index; BP—blood pressure; F. glucose—fasting glucose; HDL—high density lipoprotein cholesterol; LDL—low density lipoprotein cholesterol; Total chol.—total cholesterol; WC—waist circumference.

Values were designated as threshold, moderate, high and very high risk, with each category contributing a certain number of points to the CVD score. Threshold risk was defined for values below the 90th centile and allocated zero points. Moderate risk, where the individual is currently at increased risk, was between the 90th and 95th centile (scoring one point), high risk was assessed as between the 95th and 97.5th centile (scoring two points) and values ≥97.5th centile were categorized as very high risk (scoring three points). Given that low levels of HDL-cholesterol are a risk factor but higher levels have a protective effect, both positive and negative scores were awarded for HDL with low levels scored as 3, 2 and 1 for the ≤2.5th, 2.5–5th and 5–10th centiles respectively, and protective levels were scored −1, −2, −3, for 90–95th, 95–97.5th and ≥97.5th centiles respectively. The scores from all variables were summed to provide a multifactor CVD index with a range of −3 to 30 where negative numbers or zero would be deemed as low risk of future CVD. A score above eight indicates that risk factors such as raised blood pressure, abnormal lipids or impaired glucose metabolism, are also present in addition to obesity, as obese children would score highly on BMI and WC, which combine to a maximum score of six.

### 2.3. Dietary Intake

Dietary intake was assessed by parent report as usual intake frequency over the six months prior to each assessment using the previously validated Australian Child and Adolescent Eating Survey, a 135 item semi-quantitative food frequency questionnaire (FFQ) [34,35,36]. More extensive dietary outcomes have been reported previously [24,37]. Nutrient intakes were computed using FoodWorks version 4.00.1158 [38] and the Australian AusNut 1999 nutrient database (All Foods, Revision 17) and AusFoods (Brands, Revision 5).

### 2.4. Statistical Analysis

Counts and percentages were used to describe the distribution of the children’s biomedical data within each CVD risk score category. Medians and interquartile range (IQR) (1st–3rd quartile) were used to describe the dietary data relative to low and high CVD risk scores. The CVD risk score was dichotomized as follows: a score of 0 if CVD risk score <9; a score of 1 if CVD risk score ≥9. The value of nine was chosen as it identifies those with more than one risk factor for CVD in addition to high risk WC and BMI, similar to the IDF definition of pediatric metabolic syndrome [30]. Medians of dietary data were compared across the two CVD risk score groups using the Wilcoxon rank-sum test. Multivariate logistic regression models, with standard errors clustered by child, to estimate the strength of the association of individual CVD risk scores for the factor and each dietary measure with the multifactor CVD score while controlling for total energy intake. The dietary measures were sugar (g), total energy (kJ), protein (g), carbohydrate (g), sodium (mg), fat (g), saturated fat (g), mono- and polyunsaturated fat (g). For each explanatory variable, two models were fit, observations of boys only and observations of girls only. Odds ratios, 95% confidence intervals and *p*-values from each model were reported. All data manipulation and statistical analyses were performed in Stata version 12 MP [39]. Significance was determined at the 5% level.

## 3. Results and Discussion

Data were obtained from 56 boys and 80 girls, resulting in 285 sets of observations (121 on boys and 164 on girls), with 51 individuals (37.5%) having a single observation, with 36 (26.5%), 34 (25%) and 15 (11%) having two, three and four observations respectively. Table 2 reports the baseline data of these children.

**Table 2:** Characteristics of the children at baseline.

Variables	Boys (*n* = 44) *	Girls (*n* = 44) *	Total (*n* = 112) *
Age (years)	8.89 ± 0.8	8.53 ± 0.9	8.7 ± 0.84
BMI (kg/m^2^)	25.48 ± 3.5	25.04 ± 4.0	25.20 ± 3.8
WC (cm)	80.50 ± 9.1	76.68 ± 9.4	78.18 ± 9.4
Systolic BP (mmHg)	100.65 ± 9.3	99.08 ± 8.6	99.65 ± 8.9
Diastolic BP (mmHg)	57.32 ± 6.0	55.92 ± 5.5	56.47 ± 5.7
Fasting glucose (mmol/L)	4.22 ± 0.4	4.19 ± 0.5	4.2 ± 0.5
Insulin (mIU/L)	11.63 ± 7.0	12.05 ± 8.2	11.88 ± 7.7
Total Cholesterol (mmol/L)	4.25 ± 0.6	4.37 ± 0.7	4.32 ± 0.7
LDL (mmol/L)	2.44 ± 0.6	2.61 ± 0.6	2.54 ± 0.6
HDL (mmol/L)	1.28 ± 0.3	1.25 ± 0.3	1.27 ± 0.3
TG (mmol/L)	1.15 ± 0.6	1.11 ± 0.6	1.12 ± 0.6

* Baseline measures include only those with full data available for this time point. Data given as mean ± standard deviation.

Table 3 reports the count and percentage of observations for boys and girls in each multifactor CVD risk score category for each biomedical measure. Eighty five percent of observations of boys and 75% of observations of girls were at or above the 95th centile (intermediate to high risk) for BMI and WC as expected in this cohort. In addition, 40% of observations of boys and almost 35% of observations of girls were at or above high risk for serum triglycerides and insulin, and over 15% of all observations had low levels of HDL cholesterol. In contrast, five of the other risk factors (systolic and diastolic blood pressure, fasting glucose, total and LDL cholesterol) had 95% or more of observations in the low risk category.

**Table 3:** Count and percent of observations by CVD risk score factor level and sex.

CVD risk factor	Threshold Risk (<90th)	Moderate Risk (90th–95th)	High Risk (95th–97.5th)	Very High Risk (≥97.5th)
**Boys**				
BMI	6 (5.0%)	13 (10.7%)	13 (10.7%)	89 (73.6%)
Waist circumference	5 (4.1%)	8 (6.6%)	9 (7.4%)	99 (81.8%)
Systolic BP	117 (96.7%)	4 (3.3%)	0 (0.0%)	0 (0.0%)
Diastolic BP	120 (99.2%)	0 (0.0%)	0 (0.0%)	1 (0.8%)
Fasting glucose	119 (98.3%)	1 (0.8%)	0 (0.0%)	1 (0.8%)
Insulin	54 (44.6%)	17 (14.0%)	13 (10.7%)	37 (30.6%)
Total cholesterol	120 (99.2%)	1 (0.8%)	0 (0.0%)	0 (0.0%)
LDL	117 (96.7%)	3 (2.5%)	0 (0.0%)	1 (0.8%)
Triglycerides	59 (48.8%)	14 (11.6%)	9 (7.4%)	39 (32.2%)
	**10th–90th**	**5th–10th**	**2.5th–5th**	**≤2.5th**
HDL	70 (57.9%)	24 (19.8%)	5 (4.1%)	19 (15.7%)
		**90–95th**	**95th–97.5th**	**≥97.5th**
HDL (protective)		1 (0.8%)	2 (1.7%)	0 (0.0%)
**Girls**				
BMI	9 (5.5%)	30 (18.3%)	31 (18.9%)	94 (57.3%)
Waist circumference	20 (12.2%)	14 (8.5%)	22 (13.4%)	108 (65.9%)
Systolic BP	161 (98.2%)	2 (1.2%)	0 (0.0%)	1 (0.6%)
Diastolic BP	163 (99.4%)	0 (0.0%)	1 (0.6%)	0 (0.0%)
Fasting glucose	158 (96.3%)	2 (1.2%)	0 (0.0%)	4 (2.4%)
Insulin	82 (50.0%)	24 (14.6%)	7 (4.3%)	51 (31.1%)
Total cholesterol	159 (97.0%)	1 (0.6%)	1 (0.6%)	3 (1.8%)
LDL	155 (94.5%)	4 (2.4%)	1 (0.6%)	4 (2.4%)
Triglycerides	86 (52.4%)	19 (11.6%)	9 (5.5%)	50 (30.5%)
	**10th–90th**	**5th–10th**	**2.5th–5th**	**≤2.5th**
HDL	113 (68.9%)	17 (10.4%)	7 (4.3%)	23 (14.0%)
		**90th–95th**	**95th–97.5th**	**≥97.5th**
HDL (protective)		4 (2.4%)	0 (0.0%)	0 (0.0%)

285 observations on 136 individuals; 121 observations on 56 boys, 164 observations on 80 girls; BMI—Body Mass Index; BP—blood pressure; HDL—high density lipoprotein cholesterol; LDL—low density lipoprotein cholesterol; WC—waist circumference.

Table 4 reports median and IQR of dietary measures by low or high CVD risk score separately for boys and girls. For boys, but not girls, the median of all dietary measures was significantly higher in the high CVD risk score group (with the exception of saturated fat). For girls, there were no significant differences between the medians of high and low CVD risk score groups in any dietary measures.

**Table 4:** Median and IQR of dietary measures by CVD risk score category (<9 and ≤9) and sex.

Diet Measure	Low risk (CVD Score < 9)		High risk (CVD Score ≥ 9)		*p*-value	
	Boys	Girls	Boys	Girls	Boys	Girls
	*n = 60 (49.9%)*	*n = 89 (54.3%)*	*n = 61 (50.1%)*	*n = 75 (45.7%)*		
	Median (IQR)		Median (IQR)			
Sugars (×100 g)	1.65 (1.3–2.0)	1.64 (1.3–2.2)	2.10 (1.6–2.7)	1.67 (1.4–2.1)	<0.001	0.629
Energy (×1000 kJ)	9.87 (8.5–11.0)	9.82 (7.5–11.6)	11.45 (9.2–13.8)	9.72 (8.0–11.5)	0.004	0.936
Protein (×10 g)	9.30 (7.8–10.6)	9.06 (8.0–11.3)	10.68 (8.1–13.3)	8.98 (7.1–11.5)	0.018	0.493
Carbohydrate (×100 g)	3.24 (2.6–3.7)	2.97 (2.5–3.7)	3.63 (3.0–4.5)	3.04 (2.7–3.6)	0.004	0.936
Total Fat (×100 g)	0.74 (0.6–0.9)	0.74 (0.5–0.9)	0.84 (0.6–1.1)	0.71 (0.5–0.9)	0.047	0.941
Saturated fat (×10 g)	3.24 (2.5–3.9)	3.18 (2.2–4.1)	3.49 (2.8–4.8)	3.00 (2.3–3.8)	0.099	0.803
Monounsaturated fat (×10 g)	2.59 (2.2–3.1)	2.61 (1.9–3.2)	2.84 (2.3–3.7)	2.60 (1.9–3.4)	0.048	0.986
Polyunsaturated fat (×1 g)	8.44 (7.3–11.0)	8.71 (6.6–11.4)	10.23 (8.6–12.3)	8.43 (6.9–11.2)	0.015	0.829
Sodium (×1000 mg)	2.03 (1.6–2.3)	1.92 (1.6–2.5)	2.24 (1.8–2.7)	1.98 (1.5–2.5)	0.017	0.901

285 observations on 136 individuals; 121 observations on 56 boys, 164 observations on 80 girls. *p*-value attained using two sample Wilcoxon rank-sum test, by CVD risk score category.

**Figure 1:**
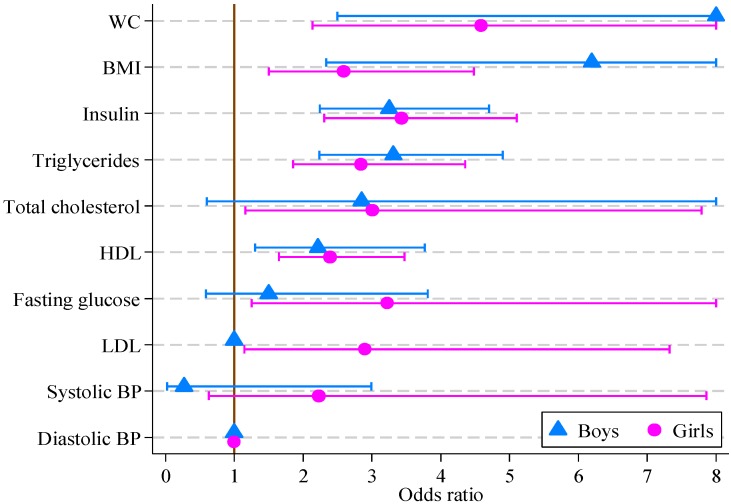
Odds ratios, with 95% confidence intervals, for boys and girls from multivariate logistic regression models of CVD risk score on individual CVD risk score factors (boys, *n* = 121; girls, *n* = 164).

The results of logistic regression modeling of CVD risk score are reported in Table 5. For girls, all factors other than systolic and diastolic blood pressure were significantly associated with the multifactor CVD risk score. For boys, the multifactor CVD risk score was associated with all factors other than blood pressure, fasting glucose, total and LDL cholesterol. The greatest odds ratios for both boys and girls were for waist circumference (14.36 and 4.59, respectively), with BMI, insulin, triglycerides and HDL also being significant. The odds ratios and 95% confidence intervals are displayed in Figure 1.

**Table 5:** Odds ratios, 95% confidence intervals (CI) and *p*-values from multivariate logistic regression models of CVD risk score on individual multifactor CVD risk score factors and total energy (e indicates total energy was significant in the model at the 5% level).

CVD Risk Score Factor	Boys (*n* = 121)			Girls (*n* = 164)		
	Odds Ratio	95% CI	*p*-Value	Odds Ratio	95% CI	*p*-Value
BMI (kg/m2)	6.20 e	2.33, 16.46	<0.001	2.59	1.50, 4.48	0.001
Waist circ. (cm)	14.01 e	2.50, 78.49	0.003	4.59	2.14, 9.83	<0.001
Systolic BP (mmHg)	0.27 e	0.02, 2.99	0.286	2.23	0.63, 7.86	0.213
Diastolic BP (mmHg)	1.00	1.00, 1.00	1.000	1.00	1.00, 1.00	1.000
Fasting glucose (mmol/L)	1.50 e	0.59, 3.81	0.395	3.23	1.25, 8.30	0.015
Insulin (mIU/L)	3.25 e	2.25, 4.71	<0.001	3.43	2.31, 5.10	<0.001
Total cholesterol (mmol/L)	2.85 e	0.61, 13.40	0.185	3.01	1.16, 7.79	0.023
LDL (mmol/L)	1.00	1.00, 1.00	1.000	2.90	1.15, 7.32	0.024
HDL (mmol/L)	2.22 e	1.30, 3.77	<0.001	2.39	1.65, 3.47	<0.001
Triglycerides (mmol/L)	3.31	2.24, 4.90	<0.001	2.84	1.85, 4.35	<0.001

The response variable is dichotomous, where 0 means CVD risk score <9 and 1 means CVD risk score ≥9 for boys and girls separately. Each explanatory variable is used to estimate the strength of its association with the multifactor CVD risk score. For example, boys increasing their waist circumference from the 90th to the 95th centile, or from the 95th to above 97.5th, are 14 times more likely to have other risk factors for CVD such as lipid or blood pressure abnormalities, in addition to a high BMI and WC.

**Figure 2:**
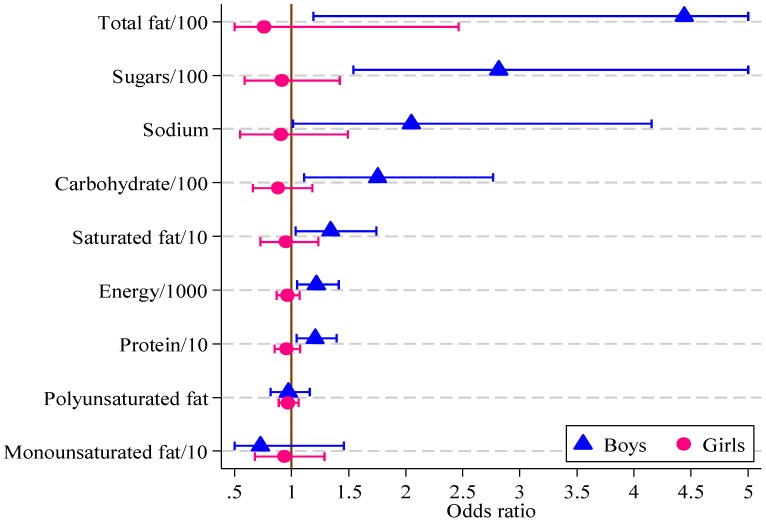
Odds ratios, with 95% confidence intervals, for boys and girls from multivariate logistic regression models of CVD risk score on diet measure (boys, *n* = 121; girls, *n* = 164).

Significant associations were found in boys between the HIKCUPS CVD risk score and all dietary measures except polyunsaturated fat (Table 6). No significant associations were demonstrated using similar models for girls, or in models using observations from both sexes, with or without the interaction between sex and dietary measures. These odds ratios and 95% confidence intervals are displayed in Figure 2.

**Table 6:** Odds ratios, 95% confidence intervals (CI) and *p*-values from multivariate logistic regression models of CVD risk score on individual diet measures and total energy (e indicates total energy was significant in the model at the 5% level).

Diet Measure	Boys (*n* = 121)			Girls (n = 164)		
	Odds Ratio	95% CI	*p*-Value	Odds Ratio	95% CI	*p*-Value
Sugars/100 (g)	2.82	1.54, 5.15	0.001	0.92	0.59, 1.42	0.696
Energy/1000 (kJ)	1.22	1.05, 1.41	0.010	0.96	0.87, 1.07	0.479
Protein/10 (g)	1.21	1.04, 1.39	0.011	0.95	0.85, 1.07	0.435
Carbohydrate/100 (g)	1.75	1.11, 2.77	0.016	0.88	0.66, 1.18	0.400
Total Fat/100 (g)	4.44	1.19, 16.60	0.027	0.76	0.23, 2.46	0.646
Saturated fat/10 (g)	1.34	1.03, 1.74	0.027	0.95	0.73, 1.23	0.690
Monounsaturated fat/10 (g)	0.73 e	0.36, 1.46	0.366	0.93	0.68, 1.29	0.678
Polyunsaturated fat (g)	0.76 e	0.13, 4.34	0.761	0.73	0.30, 1.80	0.493
Sodium/1000 (mg)	2.05	1.01, 4.15	0.047	0.90	0.55, 1.49	0.694

The response variable is dichotomous, where 0 means CVD risk score <9 and 1 means CVD risk score ≥9 for boys and girls separately. Each explanatory variable is used to estimate the strength of its association with the multifactor CVD risk score. For sugars, each increase of 100 g triples the odds of the CVD risk score being equal to or above 9.

The current study describes the development of a multifactor CVD risk score that reflects the clustering of CVD risk score factors in pre-pubertal overweight and obese children. The main factors contributing to higher CVD risk score in this pediatric group were high WC and BMI, elevated serum insulin and triglycerides, and low HDL concentrations. Elevated blood pressure was rare. Dietary measures, including high intakes of total and saturated fat, carbohydrate and sugars were significantly related to CVD risk score in boys only with no associations between a range of dietary measures and multifactor CVD risk score in girls.

The risk factors identified in the current study are not entirely consistent with other studies in overweight and obese children. Although 48% of girls and 50% of boys had triglyceride concentrations above the 90th centile in the current study, with similar results for fasting insulin levels, most had values for blood pressure, fasting glucose, total and LDL cholesterol below the 90th percentile. An Australian study of 1430 eight year olds [40] (boys and girls 15% and 20% overweight or obese, respectively) found significant differences between normal and overweight/obese participants for triglycerides, HDL, systolic and diastolic pressure. Data from the Bogalusa study [41] showed that overweight children (aged 5–10 years) had a higher prevalence of elevated insulin and triglyceride levels, as well as systolic blood pressure, total and LDL cholesterol, compared to those of normal weight (defined as >95th centile for race, age and sex, >130 mg/dL, >95th centile according to National High Blood Pressure Education Program methods, >200 mg/dL and >130 mg/dL respectively). In a sample of European overweight and obese children [13] aged 12 ± 3 years (31% pre-pubertal), elevated blood pressure was the most prevalent CVD risk score factor at 35% (assessed by height >95th centile of European reference ranges). Potential influences on the reference ranges include disparities in centile or population reference values based on age, sex, race and height; differences in assay techniques; and possibly diurnal differences in serum risk factor concentrations.

The combination of low levels of HDL with elevated serum insulin and triglyceride concentrations increases CVD risk score and was found in approximately one third of children in the current study. Insulin resistance increases triglyceride production, which in turn facilitates development of small dense LDL particles that are more susceptible to oxidation [42], further increasing CVD risk. Data compiled from four major child cardiovascular studies (Bogalusa Heart, Muscatine, Young Finns and Childhood Determinants of Adult Health) [8,43] were able to predict subclinical atherosclerosis in children aged nine years or older by identifying those with elevated total cholesterol, triglycerides, blood pressure and BMI. In addition, Lawlor *et al.* [44] and Nyugen *et al.* [28] argue that positive and rapid changes in BMI over time further increase the risk of CVD and metabolic syndrome and need to be considered when assessing risk.

Studies examining sex-based associations between diet and CVD risk in children, particularly amongst those overweight or obese, are limited and findings inconsistent making comparisons across studies difficult. A study of a large European pediatric cohort [45] found that boys aged 6 to 9 years who consumed nuts and seeds or had high intakes of chocolate and nut-based spreads had a lower CVD risk whereas girls had a higher risk in association with high intakes of manufactured juices and lower risk with chocolate and nut-based spreads. Ambrosini *et al*. [46] found 14 year old girls, but not boys, who had diets high in fat, refined sugars and sodium had greater clustering of risk factors for metabolic syndrome. In a Mexican population of a similar age [47], positive correlations were found between white bread and fasting insulin concentrations; between fasting glucose and sugar sweetened beverages and between added fats and serum triglycerides. However, no differences were reported by sex or weight status.

Sex-based responses to diet may influence CVD risk secondary to differences in hormone profiles, lipid metabolism or lifestyle behaviors, as suggested previously by Ambrosini *et al*. [46]. Differences in maturation and hormonal status of children may have influenced the results and account for some of the sex differences in this study. Reinehr and Toschke [48] found that CVD risk factors vary by stage of puberty. In the current study, pubertal status was assessed at baseline only, with follow-up time points up to two years included. Therefore it is not known whether any participants entered puberty during this time. Consequently the results need to be interpreted with some caution [48] and further investigation is warranted. Reinehr [49] suggests that future research focus on identifying which high-risk adolescents respond to specific treatment approaches, with the current study supporting a focus on dietary interventions particularly for boys shown to be at elevated CVD risk prior to puberty.

Clustering of CVD risk factors is common [5,13,41,50]. The strength of the CVD risk score developed in the current study is that it combines graded scores based on ten CVD risk variables [30,51]. Deriving a combined CVD risk score from measures of adiposity, blood lipids, carbohydrate metabolism and blood pressure referenced to normal ranges has been used in previous studies [5,17,52]. Kelly *et al*. [53] found that clustered scores were better predictors of metabolic syndrome, and that it overcame issues associated with arbitrary cut-points for some criteria [54]. The benefit of such a tool is that it is easier than calculating z-scores or quintiles, as is commonly used currently [53,55]. Using both BMI and WC allows more accurate identification of adiposity and abdominal obesity, rather than those with greater lean muscle mass.

When attempting to create a risk assessment tool, there are limitations that need to be acknowledged. The current tool would be strengthened by applying it to a larger and more varied cohort in a much longer study that followed children into adulthood to obtain objective CVD outcomes. Whilst this study used the 90th centile as the cut-point across all risk factors to ensure consistency across reference values, it is acknowledged that this may reduce sensitivity in identifying all those at increased risk secondary to high BMI. Some studies [28,44] have suggested that change in body weight may be important and this is not included in the current study. Mattsson [29] suggests that true CVD risk assessment needs to include family history of CVD and metabolic syndrome to account for genetic contributions, which were not assessed as part of the HIKCUPS study. Weight status of parents and family lifestyle behaviors could also be included in future risk assessment [4,40]. In practice, BP, BMI, WC and family history are more likely to be measured due to their low cost, whereas blood tests to assess plasma lipids are less regularly performed [56]. The use of normal reference ranges developed on other pediatric populations also has inherent difficulties. Although reported to be consistent with other Caucasian children for WC, blood pressure, serum lipids and glucose metabolism [18], the reference ranges used here were developed in French children. Variations exist when obtaining WC measures as an indicator of central adiposity in children (umbilicus, narrowest point, mid-point). This can be difficult to measure in overweight and obese pediatric participants and variations are known to exist within the measurements [57] compared to measures such as height and weight. In the current study WC measures and reference values were obtained by different methods and may have influenced the results. The use of blood pressure centiles based on height could also yield different risk scores, by allowing higher blood pressure ranges in taller children [58]. In the current study the CVD risk score for each factor was weighted as equally important but this may an over-simplification and hence results should be interpreted with caution. Future studies should investigate this further, and include additional factors associated with insulin resistance [54].

## 4. Conclusions

The assessment of CVD risk based on cut-point using greater than or equal to the top tenth percentile of published normal reference ranges of variables associated with CVD risk in overweight children has demonstrated that those with a WC or BMI at or above the 90th centile were more likely to also have other CVD risk factors present. Overweight boys with high dietary intakes of fat and carbohydrate in particular had significantly more CVD risk factors elevated above the 90th centile. Future work is needed in larger cohorts to examine the relationship of sex-specific CVD risk factors in association with dietary factors and whether this could provide opportunities for development and testing of early prevention programs targeting these factors.
